# Idiopathic isolated bilaterally symmetrical brachymetacarpia of the fifth metacarpal in a woman with rheumatoid arthritis

**DOI:** 10.1093/bjrcr/uaaf043

**Published:** 2025-08-22

**Authors:** Mohamed Tazi, Fatima Zahrae Taik, Youssef El Hassnaoui, Anass Adnine, Nihad Takrifa, Fatima Ezzahra Abourazzak

**Affiliations:** Faculty of Medicine and Pharmacy of Tangier, Abdelmalek Essaadi University, Tangier 90100, Morocco; Faculty of Medicine and Pharmacy of Tangier, Abdelmalek Essaadi University, Life and Health Sciences Laboratory, Tangier 90100, Morocco; Faculty of Medicine and Pharmacy of Tangier, Abdelmalek Essaadi University, Tangier 90100, Morocco; Faculty of Medicine and Pharmacy of Tangier, Abdelmalek Essaadi University, Life and Health Sciences Laboratory, Tangier 90100, Morocco; Faculty of Medicine and Pharmacy of Tangier, Abdelmalek Essaadi University, Life and Health Sciences Laboratory, Tangier 90100, Morocco; Faculty of Medicine and Pharmacy of Tangier, Abdelmalek Essaadi University, Life and Health Sciences Laboratory, Tangier 90100, Morocco

**Keywords:** brachymetacarpia, short metacarpal, rheumatoid arthritis, idiopathic, skeletal anomaly

## Abstract

We report a rare case of idiopathic, isolated, and bilaterally symmetrical brachymetacarpia of the fifth metacarpal bones in a 60-year-old woman followed for seropositive rheumatoid arthritis. Despite the radiographic anomaly, the patient remained asymptomatic and had normal hand function. Extensive investigations ruled out syndromic or metabolic causes. This case emphasizes the importance of recognizing rare skeletal variants in rheumatologic evaluations.

## Introduction

Brachymetacarpia refers to the congenital or acquired shortening of a metacarpal bone. It is usually observed as part of a syndrome or a post-traumatic condition but can rarely occur in isolated and symmetrical forms. Isolated bilateral shortening of the fifth metacarpals is extremely rare and often asymptomatic. In rheumatoid arthritis (RA), hand deformities are usually attributed to joint damage, but congenital anomalies should not be overlooked.

## Case report

**Figure 1.**
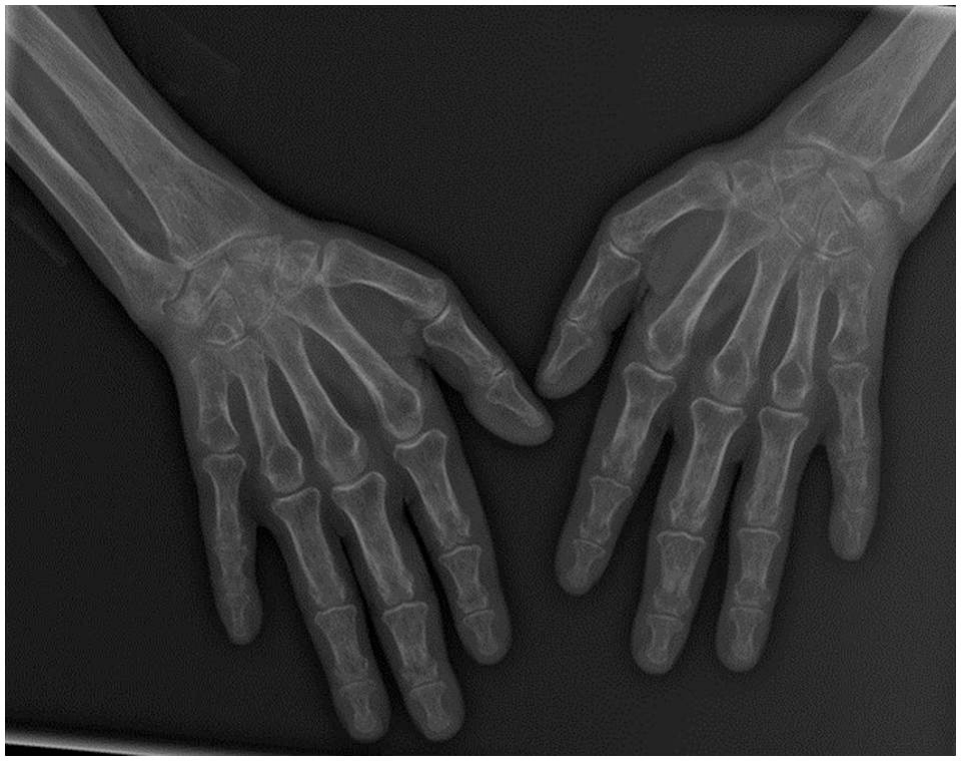
X-ray hand show an idiopathic isolated bilaterally and symmetrical brachymetacarpia of the fifth metacarpal bone.

A 60-year-old woman presented with seropositive RA, anti-cyclic citrullinated peptide antibody negative (ACPA), erosive and limiting, treated with methotrexate 25 mg/week and zoledronic acid for non-fracturing osteoporosis. She also had diffuse interstitial pneumonitis. During routine rheumatologic imaging, symmetrical shortening of both fifth metacarpals was discovered. Plain radiography of both hands demonstrated idiopathic, isolated, bilateral, and symmetrical brachymetacarpia of the fifth metacarpal bones (Figure 1).

### 1. Clinical findings

No complaints of hand weakness or deformity, normal neurological examination, and no history of trauma or family history of congenital anomalies.

### 2. Radiographic findings

X-ray both hands facing show bilateral, symmetrical shortening of the fifth metacarpals, approximately 3 cm, flattened metacarpal heads, multiple Metacarpophalangeal/Proximal Interphalangeal geodes, erosions in the left third IPP, and generalized bone demineralization.

### 3. Investigations

Serum Parathormone, calcium, phosphate, karyotype, and haemoglobin electrophoresis were within normal limits.

### 4. Functional evaluation

The grip strength was preserved, and there was no limitation in fine motor function and no aesthetic concern or history of trauma.

## Discussion

Brachymetacarpia is a rare anomaly characterized by the shortening of one or more metacarpal bones.

It may be congenital or acquired and can be either isolated or part of a broader syndrome.

Brachymetacarpia can result from several aetiologies.

### 1. Etiologies of brachymetacarpia

*Idiopathic*: Most common form, particularly when bilateral and symmetrical, with no associated systemic anomalies.^1^

*Genetic syndromes*: Often observed in Turner syndrome, pseudohypoparathyroidism, pseudopseudohypoparathyroidism, Silver-Russell syndrome, Down syndrome, and other congenital malformation syndromes.^1^^,^^2^

*Endocrine causes*: Primary hypoparathyroidism and resistance to parathyroid hormone may lead to growth disturbances of the metacarpals.^1^

*Post-infectious*: Osteomyelitis and tuberculosis dactylitis can result in growth plate damage and early closure.^3^

*Post-traumatic*: Injury during childhood may arrest metacarpal growth, leading to acquired brachymetacarpia.^3^

*Iatrogenic*: Radiation or surgery near growth plates can contribute to local growth disturbances.

*Hereditary multiple exostoses*: This rare genetic condition may also present with metacarpal shortening due to osteochondromas.^1^

In our case, the idiopathic nature is supported by normal biochemical and genetic workup and the absence of trauma or a familial history of congenital anomalies.

This finding matches the few published cases:

Schoeller et al^1^ described an idiopathic symmetrical shortening of the fifth metacarpals with no functional impairment.

Aski et al^3^ reported bilateral brachymetacarpia and metatarsia in a young female without symptoms.

### 2. Brachydactyly classification

Our patient fits into type E of Bell’s classification, involving shortening of the fifth metacarpal without phalangeal involvement or other anomalies.^2^

This type is the least likely to cause disability and often requires no intervention unless aesthetic or functional issues arise.

Importantly, in patients with RA, radiologic findings in the hands are often interpreted through the lens of disease-related joint destruction. However, the symmetry and metacarpal-specific shortening in our case, coupled with the absence of trauma, argue against RA as the primary cause. This highlights a key diagnostic challenge, distinguishing congenital skeletal anomalies from inflammatory or degenerative changes in the context of systemic rheumatic diseases.

## Conclusion

This is a rare presentation of idiopathic, isolated, bilaterally symmetrical brachymetacarpia of the fifth metacarpal bones in a woman with RA. This association remains rare and has not been reported in the literature. Recognizing such congenital anomalies is essential in rheumatologic imaging to avoid misattributing them to inflammatory joint damage and to ensure accurate clinical interpretation.

## Learning points

- *Understanding brachymetacarpia*: Brachymetacarpia is a condition characterized by the shortening of one or more metacarpal bones, affecting hand function and grip strength.
- *Case presentation*: The case highlights a patient with idiopathic isolated bilateral symmetrical brachymetacarpia of the fifth metacarpal in the context of rheumatoid arthritis (RA), emphasizing the uniqueness of this presentation.
- *Role of rheumatoid arthritis*: The case illustrates how RA can coexist with skeletal anomalies, such as brachymetacarpia, even in the absence of direct hand-related symptoms.
- *Radiological findings*: The diagnosis was primarily supported by standard radiographic findings, which revealed bilateral shortening of the fifth metacarpals and erosions in other areas, showcasing the importance of imaging in diagnosis.
- *Idiopathic nature*: In this case, the absence of trauma, normal blood test results, and lack of familial history suggest that the condition is idiopathic, which can be challenging but is essential for proper management.

## Consent

Written informed consent was obtained from the patient(s) for publication of medical information for the purpose of the publication titled “Symmetrical Brachymetacarpia of the Fifth Metacarpal Bone Isolated in a Woman Diagnosed with Rheumatoid Arthritis,” including accompanying images.
